# Supplementary material for: The Construction and Exploration of a Comprehensive MicroRNA Centered Regulatory Network in Foxtail Millet (Setaria italica L.)
Source: Front Plant Sci. 2022 May 6;13:848474. doi: 10.3389/fpls.2022.848474 (PMC9121102; doi:10.3389/fpls.2022.848474)
Supplement: Supplementary file 3 [file Data_Sheet_3.zip › Sit-miR5179_Seita.9G233700.1_5496_TPlot.pdf]

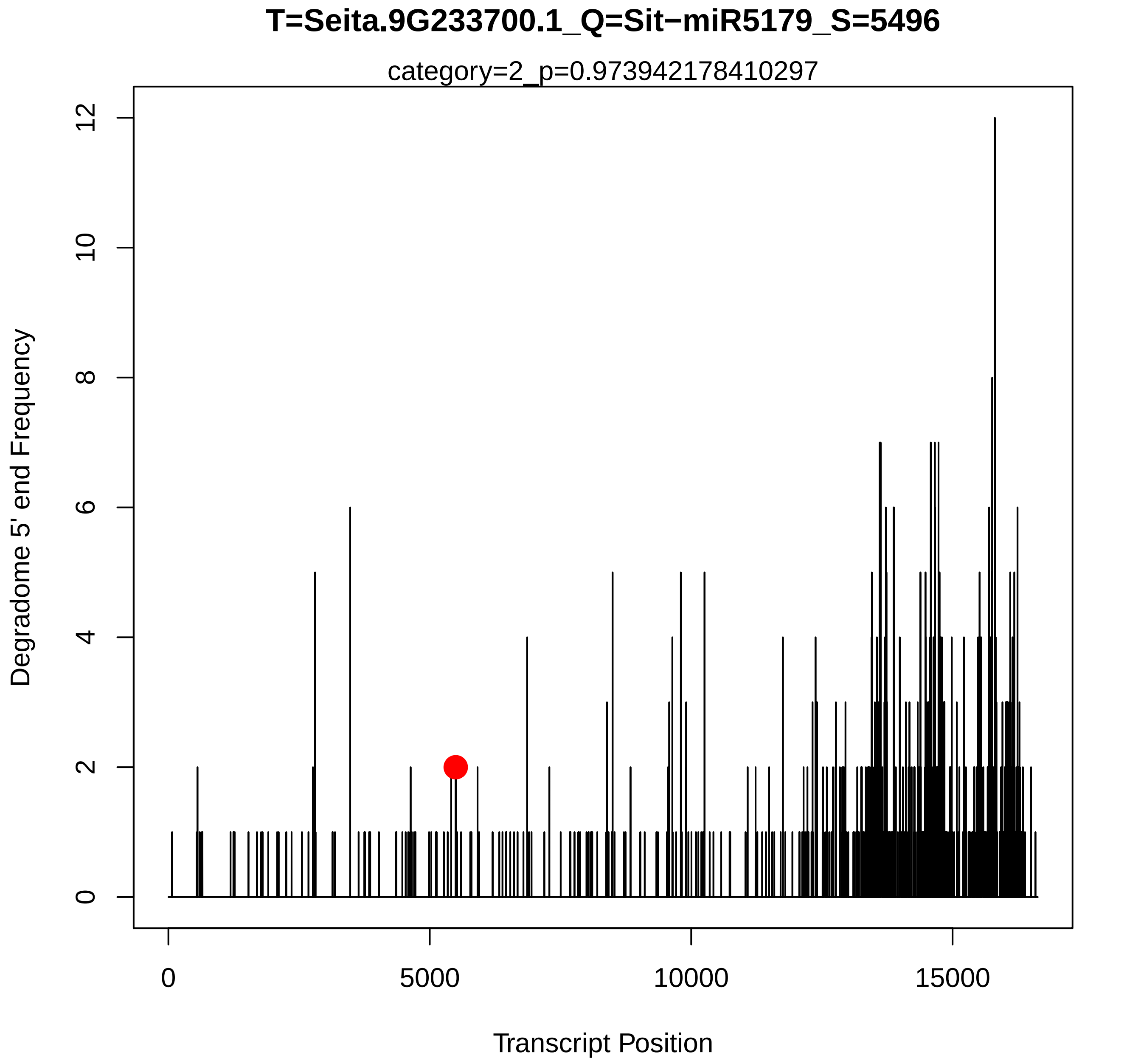
T=Seita.9G233700.1_Q=Sit−miR5179_S=5496
category=2_p=0.973942178410297
Degradome 5' end Frequency
Transcript Position
0
2
4
6
8
10
12
0
5000
10000
15000
